# SARS‐CoV‐2 induced HDL dysfunction may affect the host's response to and recovery from COVID‐19

**DOI:** 10.1002/iid3.861

**Published:** 2023-05-16

**Authors:** Hayder M. Al‐kuraishy, Nawar R. Hussien, Marwa S. Al‐Niemi, Esraa H. Fahad, Ali K. Al‐Buhadily, Ali I. Al‐Gareeb, Sadiq M. Al‐Hamash, Christos Tsagkaris, Marios Papadakis, Athanasios Alexiou, Gaber El‐Saber Batiha

**Affiliations:** ^1^ Department of Pharmacology, Toxicology Medicine College of Medicine Al‐Mustansiriyah University Baghdad Iraq; ^2^ Department of Clinical Pharmacy, College of Pharmacy Al‐Farahidi University Bagdad Iraq; ^3^ Faculty of pharmacy The University of Mashreq Bagdad Iraq; ^4^ Department of Clinical Pharmacology, Medicine and Therapeutic, Medical Faculty, College of Medicine Al‐Mustansiriya University Baghdad Iraq; ^5^ Ibin‐Al‐Bittar hospital Baghdad Iraq; ^6^ Department of Health Sciences Novel Global Community Educational Foundation Hebersham New South Wales Australia; ^7^ Department of Surgery II, University Hospital Witten‐Herdecke University of Witten‐Herdecke Wuppertal Germany; ^8^ Department of Science and Engineering Novel Global Community Educational Foundation Hebersham New South Wales Australia; ^9^ AFNP Med Austria Wien Austria; ^10^ Department of Pharmacology and Therapeutics, Faculty of Veterinary Medicine Damanhour University Damanhour AlBeheira Egypt

**Keywords:** angiotensin‐converting enzyme 2 (ACE2), cardio‐metabolic disorders, Covid‐19, dyslipidemia, high‐density lipoprotein (HDL), low‐density lipoprotein (LDL), SARS‐CoV‐2 infection

## Abstract

**Introduction:**

Covid‐19 is linked with the development of cardio‐metabolic disorders, including dyslipidemia, dysregulation of high‐density lipoprotein (HDL), and low‐density lipoprotein (LDL). Furthermore, SARS‐Co‐2 infection is associated with noteworthy changes in lipid profile, which is suggested as a possible biomarker to support the diagnosis and management of Covid‐19.

**Methods:**

This paper adopts the literature review method to obtain information about how Covid‐19 affects high‐risk group patients and may cause severe and critical effects due to the development of acute lung injury and acute respiratory distress syndrome. A narrative and comprehensive review is presented.

**Results:**

Reducing HDL in Covid‐19 is connected to the disease severity and poor clinical outcomes, suggesting that high HDL serum levels could benefit Covid‐19. SARS‐CoV‐2 binds HDL, and this complex is attached to the co‐localized receptors, facilitating viral entry. Therefore, SARS‐CoV‐2 infection may induce the development of dysfunctional HDL through different mechanisms, including induction of inflammatory and oxidative stress with activation of inflammatory signaling pathways. In turn, the induction of dysfunctional HDL induces the activation of inflammatory signaling pathways and oxidative stress, increasing Covid‐19 severity.

**Conclusions:**

Covid‐19 is linked with the development of cardio‐metabolic disorders, including dyslipidemia in general and dysregulation of high‐density lipoprotein and low‐density lipoprotein. Therefore, the present study aimed to overview the causal relationship between dysfunctional high‐density lipoprotein and Covid‐19.

## Graphical abstract

While Covid‐19 is linked with the development of cardio‐metabolic disorders, including dyslipidemia and dysregulation of high‐density lipoprotein and low‐density lipoprotein, this study aimed to overview the causal relationship between dysfunctional high‐density lipoprotein and Covid‐19.

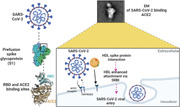

## 1 INTRODUCTION

**Figure 1:**
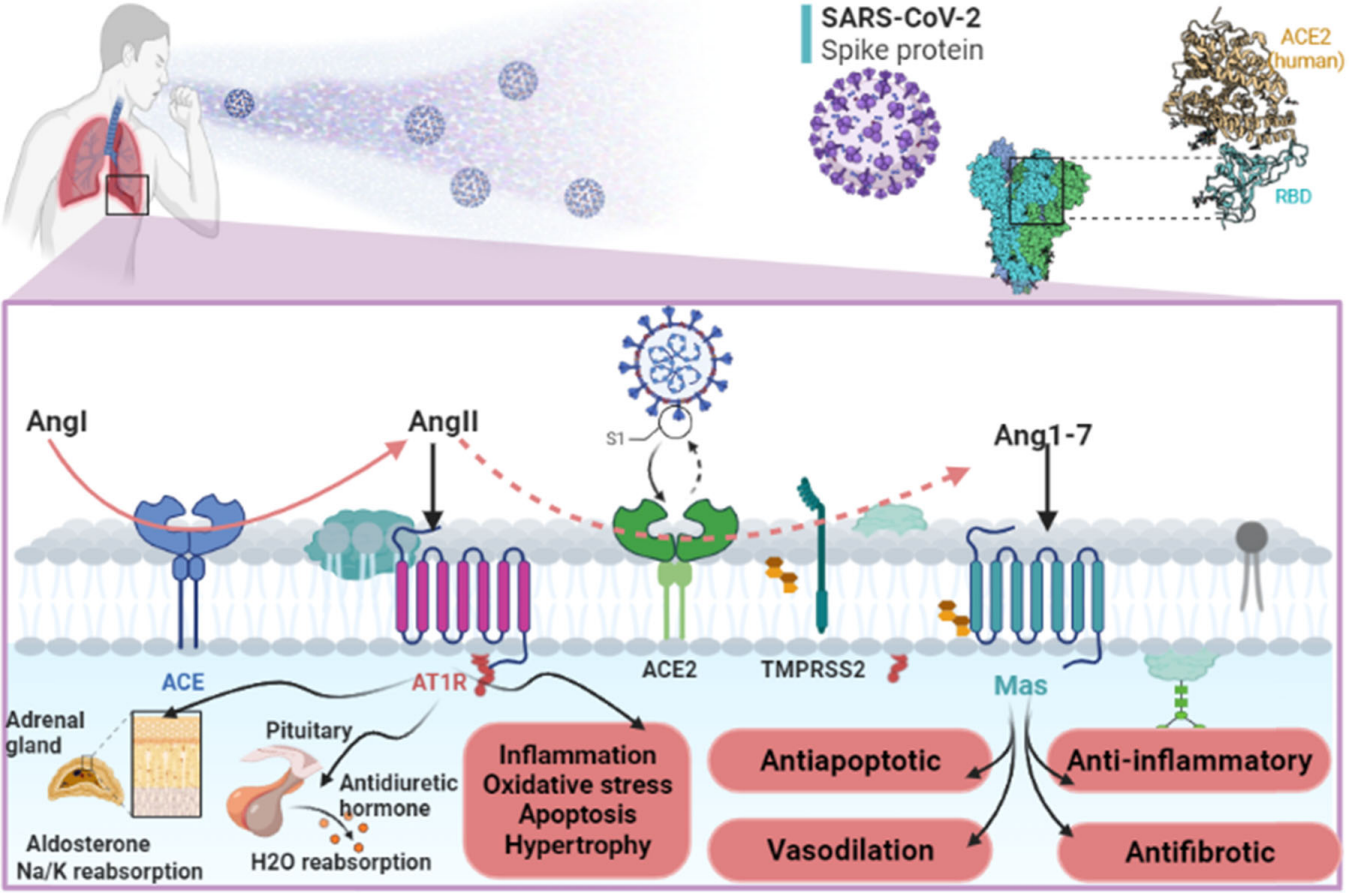
Angiotensin II (AngII) in Covid‐19: Angiotensin‐converting enzyme 2 (ACE2) as a receptor for entry for SARS‐Co‐2, downregulation of ACE2 causes over‐activation of the renin‐angiotensin system. ACE converts AngI to AngII, ACE2 converts AngII to Ang1‐7, which acts on the Mas receptor leading to protective effects. On the other hand, AngII through angiotensin type 1 receptor leads to harmful effects.

In the last of 2019, a new pandemic known as coronavirus disease 2019 (Covid‐19) emerged, causing catastrophic effects in the world.^1^ A novel virus causes Covid‐19 called severe acute respiratory syndrome coronavirus type 2 (SARS‐Co‐2), a positive sense‐single strand RNA virus from the *Betacoronaviridiae* family.^2^ The clinical presentation of Covid‐19 is asymptomatic or presented with mild flu‐like illness in 80% of cases.^3^ However, Covid‐19 in high‐risk group patients like hypertension, diabetes mellitus, cardio‐metabolic disorders, chronic kidney disease, and male sex may cause severe and critical effects due to the development of acute lung injury (ALI) and acute respiratory distress syndrome (ARDS).^3^ Of note, Covid‐19 may lead to extra‐pulmonary manifestations, including stroke, acute kidney injury, acute hepatic injury, acute cardiac injury, testicular failure, and various forms of endocrinopathies.^4^ SARS‐Co‐2 exploits angiotensin‐converting enzyme 2 (ACE2) as a receptor for entry to the host cells. ACE2 is highly expressed in immune cells, cardiomyocytes, renal tubules, enterocytes, testicular cells, and lung alveolar cells.^5^ ACE2 is responsible for the metabolism and conversion of vasoconstrictor angiotensin II (AngII) to the vasodilator Ang1‐7. Downregulation of ACE2 by SARS‐Co‐2 augments the elevation of AngII, which has pro‐inflammatory and proliferative effects (Figure 1).^6^

Covid‐19 is linked with the development of cardio‐metabolic disorders, including dyslipidemia in general and in a particular dysregulation of high‐density lipoprotein (HDL) and low‐density lipoprotein (LDL).^7^ Different human studies illustrated that SARS‐Co‐2 infection is linked with significant changes in lipid profile, which was suggested as a possible biomarker to support the diagnosis and management of Covid‐19.^7^ Many studies highlighted that reduction of HDL in Covid‐19 is connected to its severity and poor clinical outcomes.^8^ Interestingly, Ding et al.^9^ revealed that low HDL reduces SARS‐Co‐2 clearance. It has been shown that low HDL serum level was meaningfully associated with a longer clearance time of SARS‐Co‐2, nearly 35.5 days from onset of Covid‐19.^9^ The expected average time from onset of Covid‐19 symptoms to the negative test for SARS‐Co‐2 infection is 9–11 days.^10^ These findings suggest that high HDL serum levels could benefit symptomatic Covid‐19. Reduced HDL levels also trigger the release of pro‐inflammatory cytokines and correlate with inflammatory markers like C‐reactive protein (CRP).^11^

In general, HDL has anti‐inflammatory improves endothelial function; however, dysfunctional HDL loses its anti‐inflammatory properties and becomes pro‐inflammatory and proatherogenic, causing endothelial dysfunction (ED) and increased risk of cardio‐metabolic disorders.^12^, ^13^ Therefore, the present study aimed to overview the causal relationship between dysfunctional HDL and Covid‐19.

## 2 MAIN TEXT

### 2.1 General characteristics of HDL

**Figure 2:**
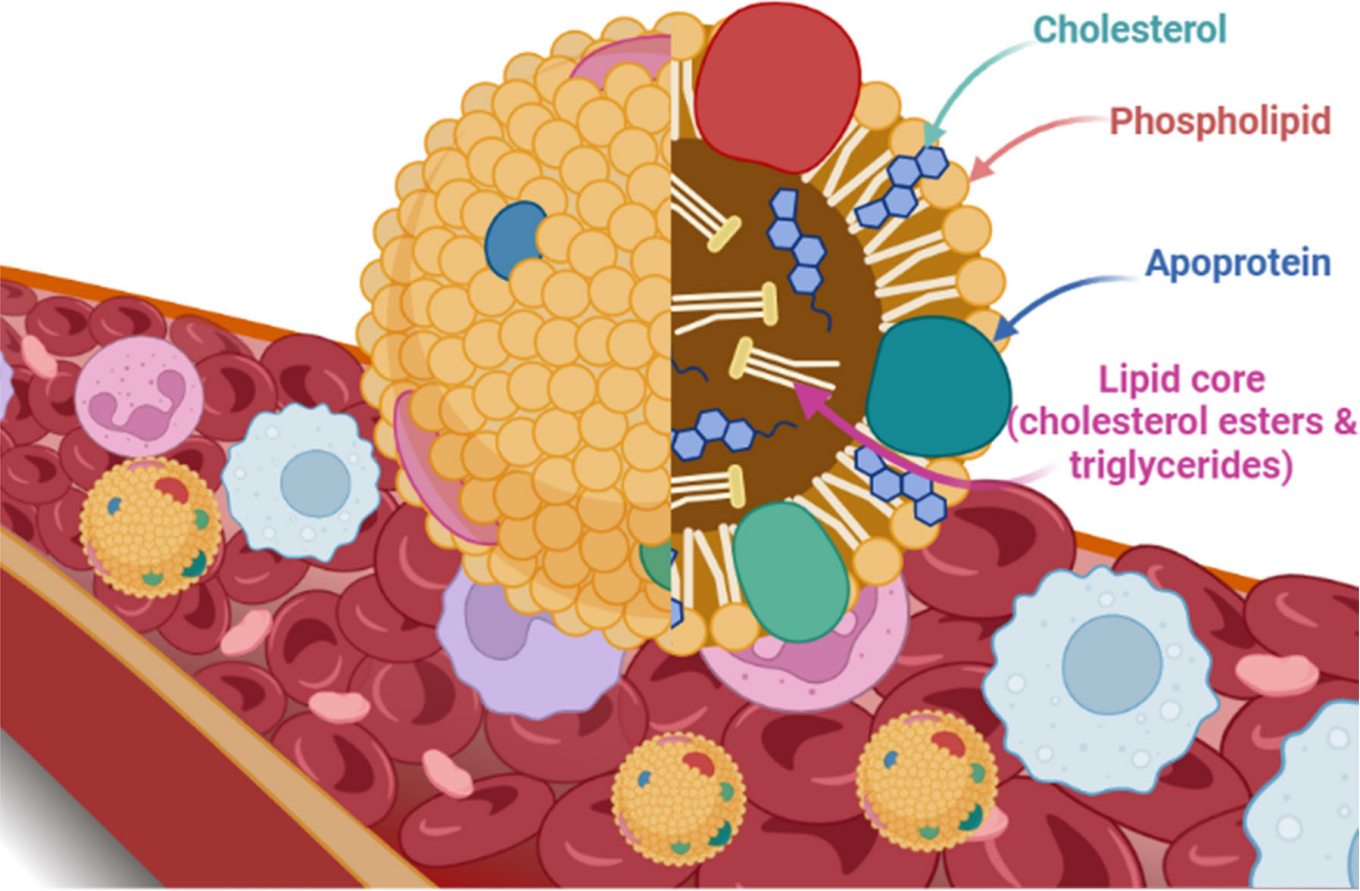
Structure of high‐density lipoprotein.

HDL is a small size particle (7–14 nm) in diameter with high density (1.06–1.21 g/mL) and specific apolipoprotein constituent.^14^ HDL is involved in multiple functions; including immunity, inflammation, proteolysis, homeostasis, and reverse cholesterol transport (RCT).^15^ These diverse functions are closely related to the specific HDL‐subspecies, though the mechanisms related to the subspecies are not elucidated.^15^ HDL is composed of a hydrophobic lipid core surrounded by free cholesterol monolayer and phospholipid studded by proteins. Apolipoprotein A1 (ApoA1) forms 70% of its protein contents (Figure 2).^15^

HDL is the smallest and densest lipoprotein that contains distinct proteins, lipid species, and nonpolar cargo molecules.^16^ HDL contains phospholipids, un‐esterified cholesterol, sphingomyelin, and triglyceride. HDL contains sphingosine‐1‐ phosphate, plasmalogens, ceramide, free fatty acids, and bioactive steroids like estrogen and oxysterols that give the functional diversity of HDL.^16^

About 95 distinct proteins and many hundreds of lipid subtypes in HDL mediate its pleiotropic effects.^16^ Kluck et al.^17^ reported that HDL carries vitamin E and other lipid‐soluble vitamins and macromolecules like microRNA. The biosynthesis of HDL started as a lipid‐free protein with ApoA1 that acquires cholesterol and phospholipids in the circulation via ATP binding cassette transporter to form premature HDL particles.^18^ Through lecithin cholesterol acyltransferase (LCAT), these particles maintain the accumulation of cholesterol and form a hydrophobic core with HDL particles.^18^, ^19^ HDL injects more cholesterol and exchanges lipids with lipoproteins via cholesteryl ester transfer protein (CETP) and phospholipids.^20^

**Figure 3:**
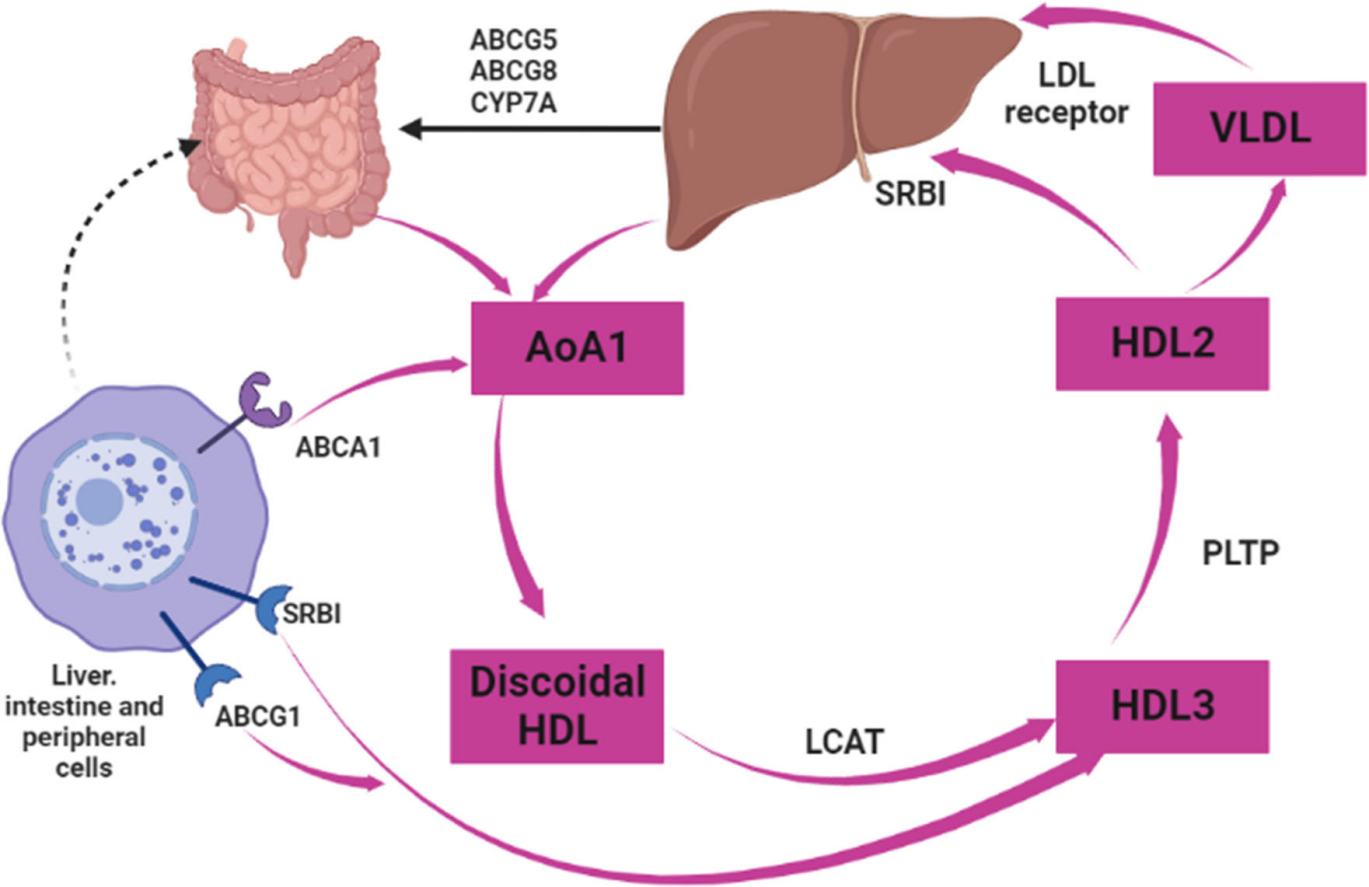
Metabolic pathway of high‐density lipoprotein (HDL): The biosynthesis of HDL started when ApoA1 acquired cholesterol and phospholipids in the circulation via ATP binding cassette transporter to form premature HDL particles. Through lecithin cholesterol acyltransferase, these particles maintain the accumulation of cholesterol and form a hydrophobic core with HDL particles. HDL takes more cholesterol in circulation and exchanges lipids with lipoproteins via cholesteryl ester transfer protein and phospholipids.

The liver takes up HDL cholesterol (HDL‐c) through selective lipid uptake without lipoprotein degradation. As a result, the liver metabolizes the cholesterol, and free HDLs are re‐circulates again (Figure 3). Similarly, selective lipid uptake from HDL is also done by steroidogenic cells in the testes, adrenal gland, and ovary.^21^

### 2.2 Pleiotropic effects of HDL

**Figure 4:**
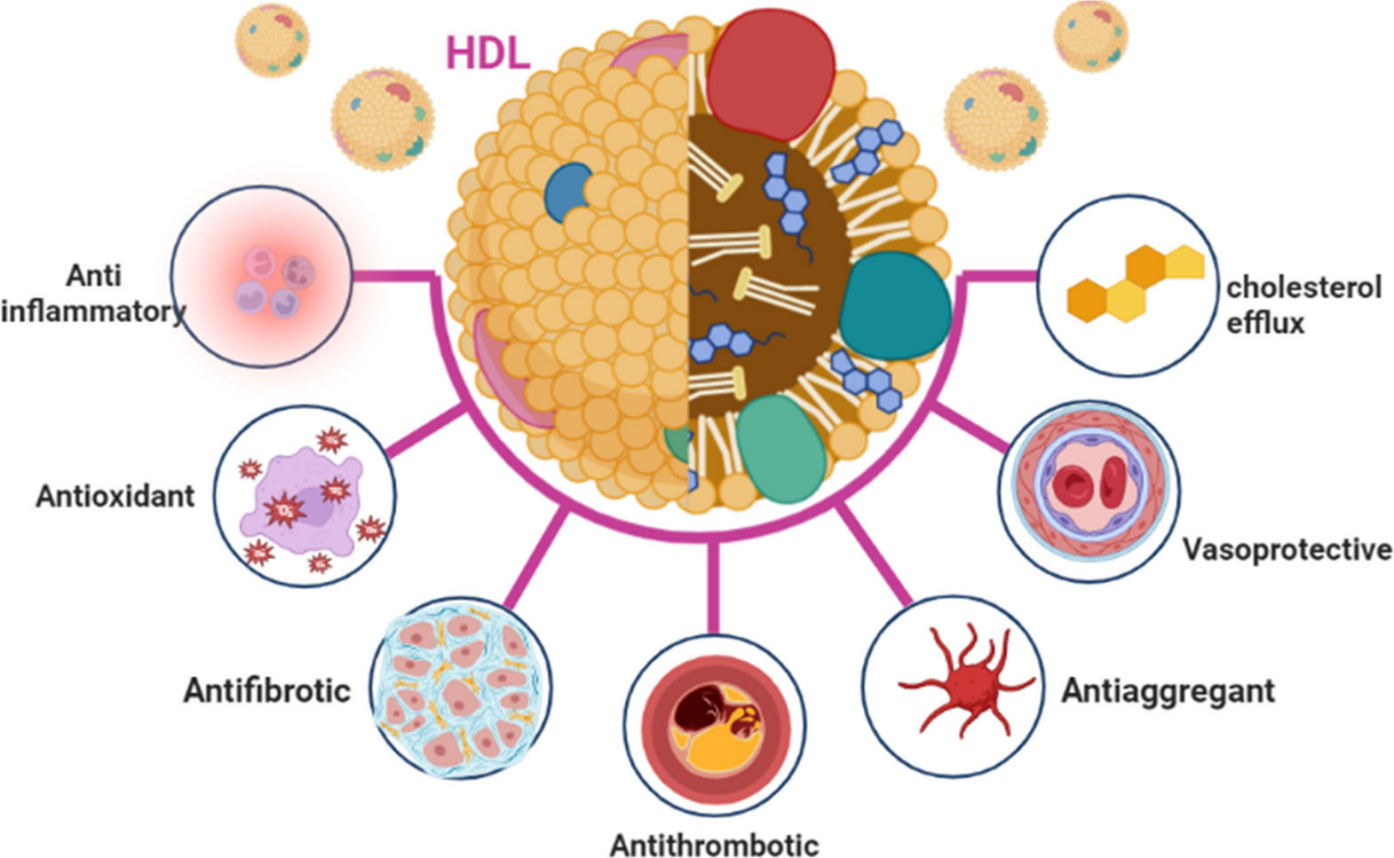
Pleiotropic effects of high‐density lipoprotein.

HDL subclasses and components vary between individuals and are altered by different diseases.^22^ It has been shown that, unlike humans, where LDL is the primary circulating lipoprotein, HDL's chief plasma protein in mice renders them resistant to cardiovascular diseases (CVD).^23^, ^24^ HDL's cholesterol efflux capacity (CEC) is affected by different diseases, including CVDs, inflammatory disorders, and metabolic syndrome, as CEC can be reduced independent of HDL.^25^ Of note, small HDL particles promote CEC through the ABCA1 transporter,^25^ suggesting that improving CEC reduces the risk of CVDs. Reduced CEC of HDL in different inflammatory disorders might be due to reduced HDL paraoxonase‐1 (PON‐1) activity or increased content of associated serum amyloid A (SAA) in HDL.^26^ Interestingly, HDL and RCT functions have many pleiotropic functions, including antioxidant, anti‐inflammatory, antiapoptotic, anticytotoxic, and vasodilatory functions (Figure 4).^27^

The anti‐inflammatory effect of HDL is through receptors‐dependent mechanisms. HDL activates scavenger receptor B1 (SR‐B1) and sphingosine‐phosphate receptor (S1P), causing the release of endothelial nitric oxide (NO).^28^ This change by HDL reduces endothelial permeability, expression of adhesion molecules, and inhibition of inflammatory signaling pathways, including nuclear factor kappa B (NF‐κB).^29^ In addition, HDL improves the expression of anti‐inflammatory annexin‐1, reduces endothelial exocytosis, and maintains endothelial nitric oxide synthase (eNOS).^30^ Furthermore, HDL PONs attenuates oxidative stress induced‐apoptosis and release pro‐inflammatory cytokines.^31^ PONs are synthesized by hepatocytes and secreted into circulation, where they assemble with HDL. PONs involves the metabolism of different agents, including lactones, thiolactones, glucuronide drugs, aryl esters, organophosphorus, nerve gases, and cyclic carbonates.^32^ Furthermore, PON‐1 can reduce myeloperoxidase activity and modulate the antioxidant activity of LCAT.^32^ Besides, platelet‐activating factor acetylhydrolase (PAF‐AH) which also called called Lp‐PLA2, and LCAT also contribute to the antioxidant effects of HDL.^19^, ^33^, ^34^

Furthermore, HDL attenuates LDL‐induced macrophages apoptosis and release of IL‐1β and tumor necrosis factor‐alpha (TNF‐α), inhibiting the expression of endothelial‐leukocyte adhesion molecules.^35^ Muid et al.^35^ in vitro study demonstrated that HDL inhibits most expression of adhesion molecules except vascular cell adhesion molecule 1(VCAM‐1). S1P and ApoA1 mainly mediate this process as most of the circulating S1P is carried by HDL.^36^

It has been shown that HDL via S1P receptor creates potent anti‐inflammatory effects through induction of macrophage polarization and chemotaxis and inhibition of endothelial inflammation.^36^ Similarly, HDL through ApoA1 induces anti‐inflammatory effects by reducing the release of inflammatory cytokines.^37^ Furthermore, an experimental study by Guo and colleagues found that deficiency of ApoA1 in mice increases the release of pro‐inflammatory cytokines. In contrast, overexpression of ApoA1 or the use of mimetic peptides reduces the risk of inflammation via neutralization of bacterial endotoxin in septic mice.^38^ These observations suggest the protective effects of HDL against inflammatory disorders through PONs, S1P, and ApoA1‐dependent pathways.

In addition, CETP improves the anti‐inflammatory effects of HDL via activation of PONs, S1P, and ApoA1, since activation of CETP reduces sepsis‐induced inflammation, increases HDL, and improves survival in experimental studies.^39^ A Cohort‐observational study involving 25 patients with heart failure showed that CETP level was low in patients with severe heart failure,^40^ suggesting CETP's protective effect against aggravation and severity of heart failure. Blauw et al.^41^ illustrated that CETP expression reduces systemic inflammation in mice. It has been reported that deficiency of CETP may alter HDL's functional capacity through modification of HDL's lipid composition, mainly the antiatherogenic one.^42^ A study comprised eight patients with CETP deficiency compared to eight healthy controls illustrated that CETP deficiency increases HDL's atherogenic lipid content and increases the risk of atherogenicity.^43^ These findings proposed the protective effects of CETP. However, expression of CETP in the endothelial cells may cause ED through upregulation of vascular cell adhesion molecule‐1 (VCAM‐1), intracellular cell adhesion molecule‐1 (ICAM‐1), and monocyte adhesion, which might contribute to the pathogenesis of atherosclerosis.^43^

**Figure 5:**
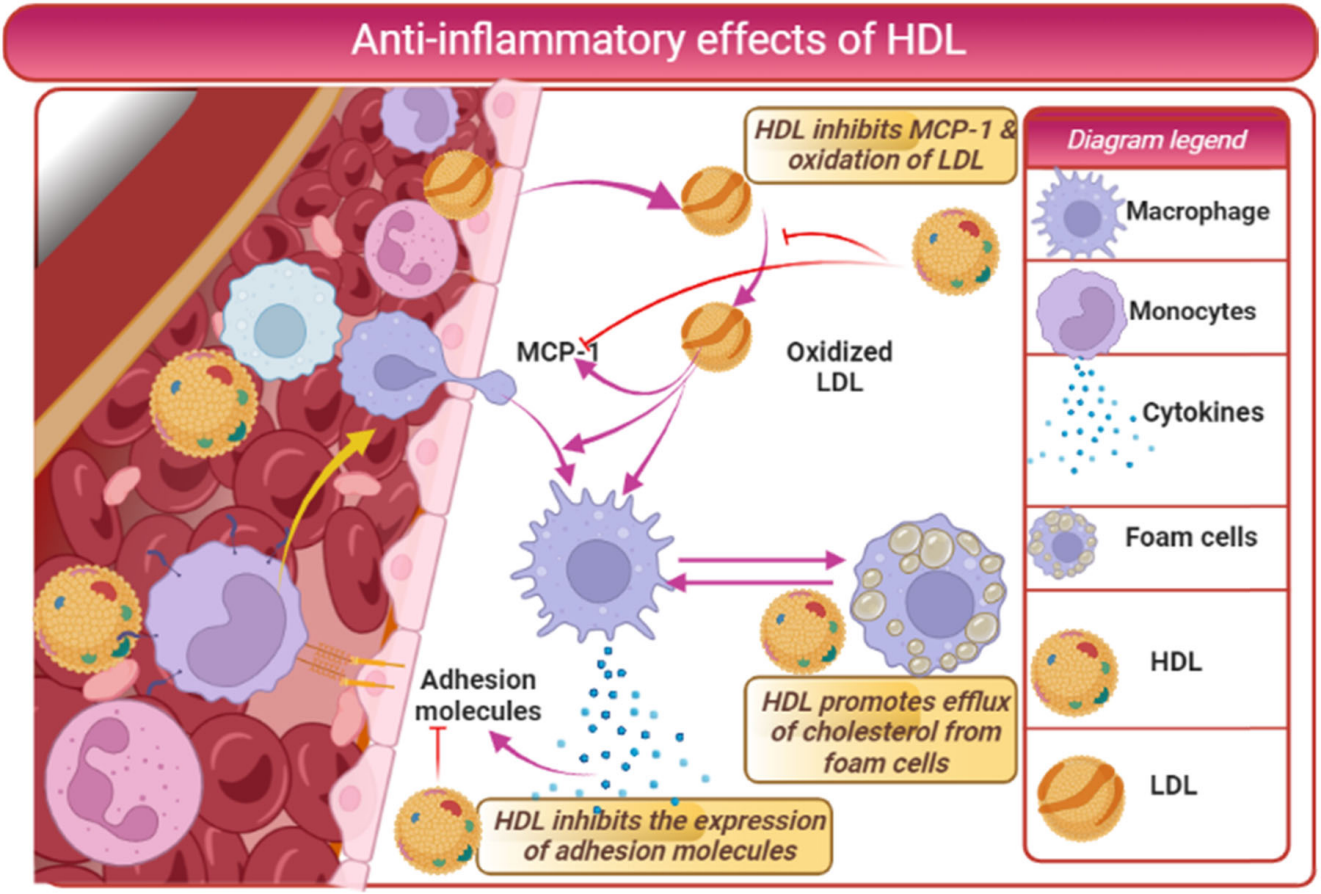
Anti‐inflammatory effects of high‐density lipoprotein (HDL): HDL inhibits expression of adhesion molecules and monocyte chemoattractant protein 1 (MCP‐1), reducing LDL oxidation and forming oxidized LDL. HDL attenuates the stimulatory effects of oxLDL on macrophages to release inflammatory cytokines.

Furthermore, HDL inhibits LDL oxidation through antioxidants ApoA1, ApoM, ApoE, and PON‐1.^44^ Cedo et al.^45^ observed that HDL attenuates LDL oxidation in patients with diabetes mellitus. Moreover, HDL inhibits the expression of adhesion molecules and monocyte chemoattractant protein 1 (MCP‐1), reducing LDL oxidation and the formation of oxidized LDL. HDL attenuates the stimulatory effects of oxLDL on macrophages to release inflammatory cytokines^45^ (Figure 5).

### 2.3 Dysfunctional HDL and inflammations

It has been reported that many diseases can affect the protective effects of HDL. For example, HDL isolated from patients with CVDs showed reduced ability to activate and phosphorylate eNOS with abnormal immune reactivity.^46^ Similarly, chronic kidney diseases reduce the protective effect of HDL through impairments of anti‐inflammatory, antioxidant, and RCT functions of HDL.^47^ Indeed, in an aortic aneurysm, circulating level of small‐HDL is reduced due to sequestration of anti‐inflammatory ApoA1 at the site of inflammation in the thrombotic area of the aneurysm.^48^ These changes that reduce HDL's anti‐inflammatory and antioxidant ability predisposes to atherogenicity and pro‐inflammatory status.^48^ Remarkably, HDL from patients with atherosclerosis exhibits dysfunctional properties even with normal HDL levels.^49^ Restoration of HDL in this condition may adversely impact CVDs and cause more pro‐atherogenic effects.^49^

Under certain inflammatory circumstances, HDL loses its athero‐protective function with subsequent induction release of pro‐inflammatory cytokines and reduced cholesterol efflux from macrophages.^50^ In addition, myeloperoxidase‐induced oxidation of ApoA1 creates dysfunctional HDL with subsequent increased risk of CVD events.^51^ There is a close link between systemic inflammation and the development of dysfunctional HDL in various CVDs.^52^ High systemic inflammation and HDL levels decrease flow‐mediated dilation since a very high HDL level is regarded as an independent risk factor for high mortality in a population‐based study.^53^

Likewise, high serum amyloid ‐A (SAA) in patients with type 1 diabetes mellitus (T1DM) impairs HDL function by which increases atherosclerotic risk.^54^ High pro‐inflammatory cytokine with high dysfunctional HDL predisposes to the risk of infections, as it acts as a mediator of inflammation during infections.^55^ In T1DM patients, the CEC of HDL is highly impaired, leading to dysfunctional HDL development and an increased risk of CVD complications.^55^ These findings suggest that HDL modification by inflammation augments the progression of the atherogenic phenotype of HDL with future cardio‐metabolic changes.

Accumulating evidence from a large body of published literature showed that chronic inflammatory disorders increase the risk of CVD events due to changes in HDL function and structure rather than its level.^56^ The exact mechanism related to HDL dysfunction is a reduction of ApoM, ApoA1, and LCAT activity with augmentation of SAA, endothelial secretory phospholipase A2, and endothelial lipase.^56^ These structural changes induce dysfunctional disorders of HDL to reduce LDL oxidation and RCT.

de‐la Liera et al.^57^ in vitro study demonstrated that endotoxemia and inflammation directly affect HDL function due to impairment of RCT independent of HDL plasma level and ApoA1 activity. Therefore, the functional properties of HDL rather than its circulating level predicts and provide more relevant information regarding CVD complications with underlying chronic inflammatory disorders.^58^ Of interest, HDL antioxidants maintain the anti‐inflammatory effects of HDL. However, in the presence of inflammation, the HDL accumulates oxidized proteins and lipids, making it pro‐inflammatory and proatherogenic, causing harmful rather than protective effects.^59^

**Figure 6:**
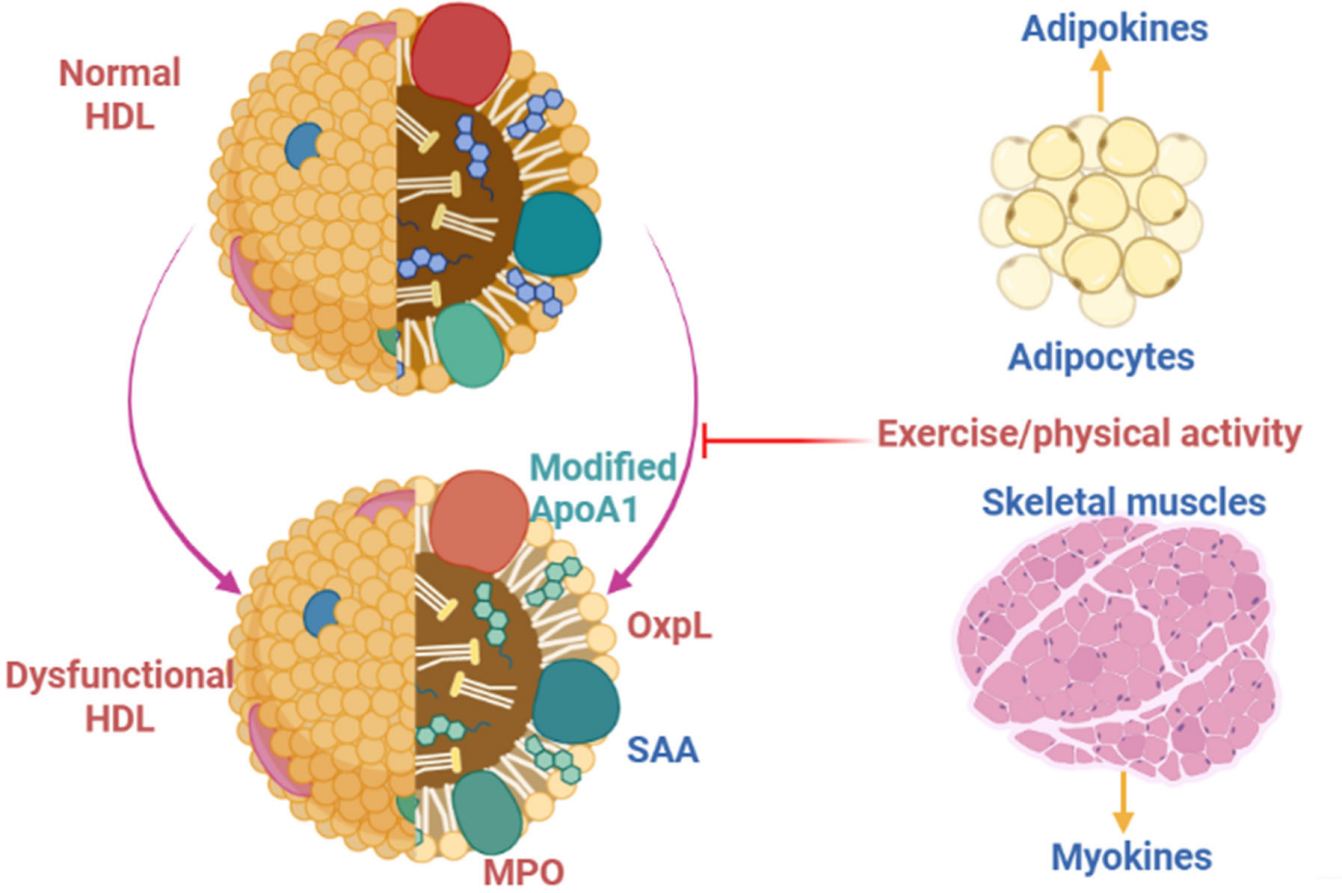
Development of dysfunctional high‐density lipoprotein (HDL): Adipokines from adipocytes and physical activity improvement promote normal HDL. At the same time, risk factors for CAD may induce modification of composition and function of HDL, causing dysfunctional HDL due to modifications of ApoA1, paraoxonase‐1, and phospholipase.

It has been proposed that proatherogenic HDL is more evident in patients with coronary heart diseases. Proteomic analysis revealed that the severity of coronary heart diseases is more related to the functional profile of HDL than its steady‐state.^60^ Further, Schill et al.^29^ illustrated that reactive oxygen species (ROS) could modify sterols and phospholipids of HDL, reducing its antioxidant capacity with the progression of oxidative stress. Animal models of vascular inflammation and dyslipidemia observed that attenuating inflammatory burden and oxidative stress may reverse dysfunctional HDL. One way was that use of ApoA1 mimetic peptide, which improves HDL's anti‐inflammatory and antioxidant properties with further attenuation of atherosclerosis progression.^29^ Therefore, chronic inflammatory disorders and oxidative stress impair HDL's anti‐inflammatory and antioxidant properties. Adipokines from adipocytes and improved physical activity promote normal HDL, while risk factors for CAD may induce modification of composition and function of HDL, causing dysfunctional HDL^29^, ^58^ (Figure 6).

### 2.4 Dysfunctional HDL in infections and immune disorders

It has been reported that alteration and modification of HDL level and function can affect the immune response. During sepsis, bacterial cell membrane components, including lipopolysaccharide (LPS), may induce an exaggerated immune response, cytokine production, and reduction of HDL levels.^61^ Thus, high HDL levels in transgenic mice improve sepsis survival.^61^ Interestingly, HDL increases the clearance of LPS via SR‐B1, which acts as an LPS‐binding protein.^41^ Therefore, low HDL is negatively correlated with sepsis and augments systemic inflammatory reactions as HDL‐SR‐B1 acts as direct anti‐inflammatory agent‐driven cholesterol for glucocorticoid synthesis.^62^, ^63^ Van‐Leeuwen and colleagues observed that HDL serum levels rapidly declined in patients with severe sepsis,^64^ suggesting a protective role of HDL against bacterial sepsis.

**Figure 7:**
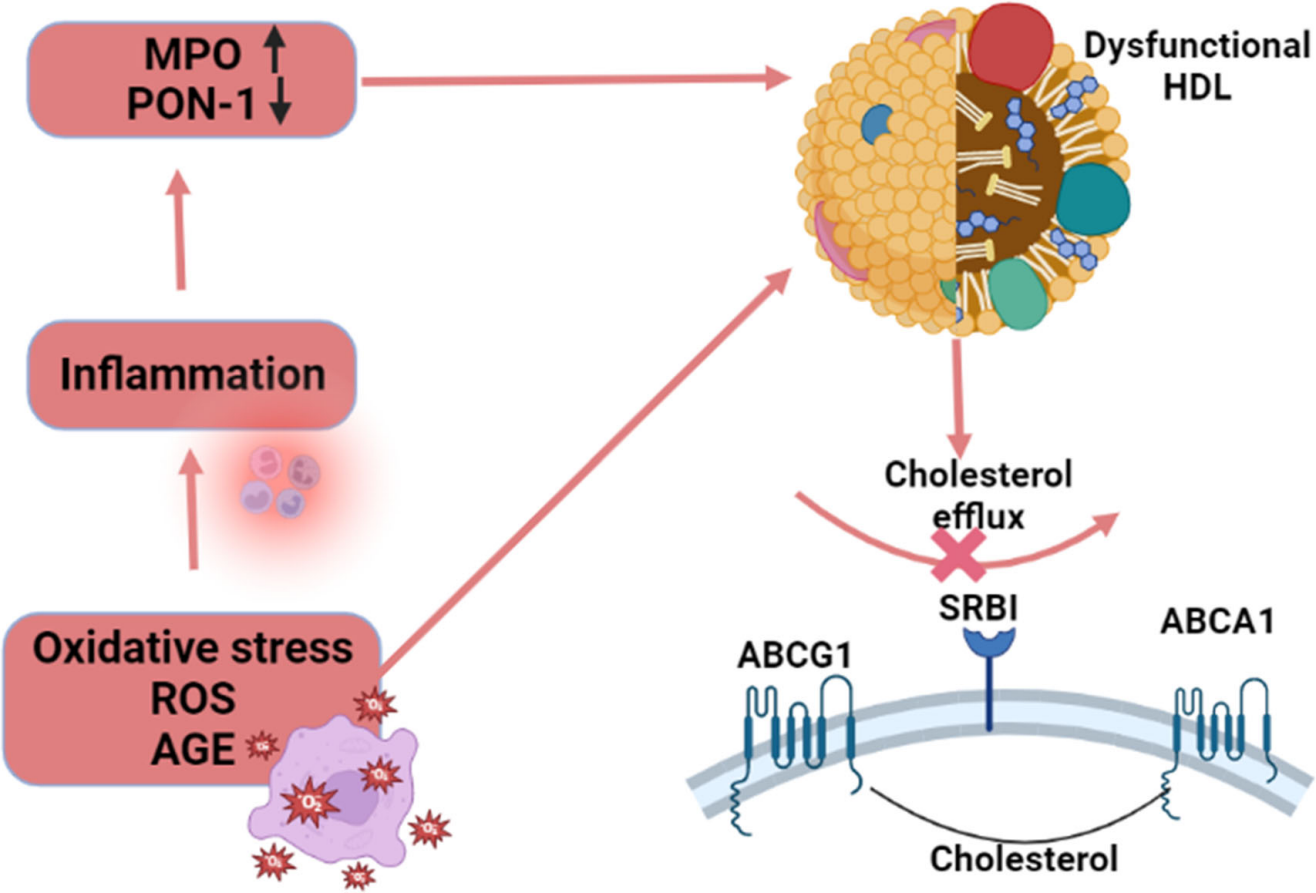
Inflammation, oxidative stress, and development of dysfunctional high‐density lipoprotein (HDL): High oxidative due to reactive oxygen species and advanced glycation end product increase inflammation, which decrease paraoxonase‐1 and increase myeloperoxidase, causing dysfunctional HDL, which lost its cholesterol efflux capacity.

Furthermore, it was observed that administration of reconstituted HDL can attenuate LPS‐induced inflammation^64^ proposed that HDL could be a valuable therapeutic approach for sepsis. Besides, intracellular bacteria and host oxidized phospholipids attenuate HDL's immune response and protective immunomodulatory effect, causing dysfunctional HDL without scavenger effect against oxidized phospholipids as in leprosy.^65^ Moreover, modifications of HDL‐specific proteins like ApoL‐1, associated with a serum complex known as trypanosome lytic factor‐1 (TLF‐1), may increase parasitic infections.^66^ Thus, high oxidative changes due to ROS and advanced glycation end product (AGE) increase inflammation which decreases PON‐1 and increases myeloperoxidase (MPO), causing dysfunctional HDL, which loses its CEC (Figure 7).

Of interest, reduction of HDL serum level is associated with the development of auto‐immune disorders. It has been shown that immune cells, including lymphocytes and dendritic cells, showed auto‐immune phenotypes in mice with deficiency of HDL.^67^ The deficiency of SR‐B1in mice induces abnormal lymphocyte proliferation and uncontrolled release of pro‐inflammatory cytokines from macrophages with subsequent deposition of immune complexes in the glomeruli and high circulating autoantibodies.^68^ Likewise, SR‐B1 null mice have a large‐size HDL particle with high cholesterol content resulting in minimal inhibitory effects on lymphocyte proliferation.^69^ In this bargain, injection of ApoA1 prevents lymphocyte activation and can reduce cholesterol content in the HDL and lymph nodes.^69^ These findings suggest that HDL plays a crucial role in infections and auto‐immune response; since abnormal immune responses may affect the functional properties of HDL.

Of note, acute phase response and immune disorders modify HDL constituents and size with noteworthy reduction of circulating HDL level, causing the development of dysfunctional HDL due to a reduction half‐life of ApoA1.^66^ These changes increase the risk of poor clinical outcomes with high mortality in cases of sepsis and endotoxemia.^70^ Furthermore, in acute phase conditions, ApoA1 of HDL is displaced by secretory phospholipase A2 and SAA, prone HDL for hepatic metabolism with a further reduction of its half‐life.^71^ Indeed, PON‐1 of HDL is reduced while PAF‐AH is increased in acute infections and inflammatory disorders that damp the antioxidant capacity of HDL.^72^

Another essential key protein present in HDL linked with acute phase response is ApoM, which binds S1P and mediates the protective effect of HDL. However, ApoM is reduced during acute inflammation and infections, resulting in abnormal HDL distribution.^73^ Thus, alteration of the ApoM/S1P axis results in the progression of dysfunctional HDL during acute inflammatory disorders.

Furthermore, dysfunctional HDL is linked with various auto‐immune disorders due to alteration in HDL and its anti‐inflammatory constituents.^68^ Feng et al.^68^ found that deficiency of SR‐B1 induces impairment of lymphocyte homeostasis and the development of auto‐immune disorders. SR‐B1 is a regulatory key of adaptive immunity that controls lymphocyte proliferation and cytokine production. It has been shown that lupus dyslipidemia, characterized by low and abnormal composition of HDL resulting in impairment of antioxidant and anti‐inflammatory properties of HDL.^74^ Also, a premenopausal woman with systemic lupus erythematosus (SLE) has a higher proportion of small HDL with dysfunctional HDL,^75^ which may predispose them to develop SLE's atherosclerosis.

Of note, type 2 diabetes mellitus (T2DM), which was re‐classified as an auto‐immune disorder rather than an only metabolic disease, displayed abnormal HDL function and metabolism.^76^, ^77^ HDL protects pancreatic β‐cells function from dyslipidemia‐induced inflammation and oxidative stress. The protective effects of HDL are reduced in T2DM due to prolonged hyperglycemia.^77^ Dysfunctional HDL in T2DM could be a consequence of metabolic derangements, and this abnormal HDL may adversely affect pancreatic β‐cells function.^77^ These observations suggest a potential role of dysfunctional HDL in the pathogenesis of T2DM.

Despite broad observations and findings of altered HDL composition in various immune disorders, whether HDL represents and symbolizes a bystander effect of immuno‐inflammatory disorders or a potential key player was unidentified.^78^ Different experimental studies revealed that HDL acts as a reservoir and pool for a series of lipids and proteins with immunomodulatory effects.^79^

HDL has a binding immunological effect, and it influences the activation of immune cells through modulation of cholesterol content in lipid rafts and expression of immune receptors.^80^ HDL and ApoA1 attenuate monocytes/neutrophils recruitment and activation by reducing monocyte adhesion to endothelial cells.^81^ Moreover, HDL triggers the polarization of macrophages from classical pro‐inflammatory M1 to the alternative anti‐inflammatory M2 macrophage, which improves the release of anti‐inflammatory cytokines in animals.^82^ These findings have not been proven in human studies since HDL did not affect M2 differentiation, and monocytes are differentiated similarly in subjects with normal and low HDL levels.^83^ Lee et al.^84^ illustrated that HDL inhibits M1 macrophages polarization through redistribution of caveolin‐1. These findings could be related to some markers of M2 not found in humans.

ApoA1 is the main functional and structural protein of HDL and plays a critical role in cholesterol efflux and, together with sphingolipids, form lipid rafts.^85^ Lipid composition of lipid rafts may alter immune response due to B cell and T cell receptors and toll‐like receptors (TLRs).^86^ In addition, ApoA1 reduces the abundance of lipid rafts in the plasma membrane of monocytes by cholesterol efflux, so it attenuates pro‐inflammatory signaling by altering the cholesterol contents of monocytes and lymphocytes.^86^

Furthermore, ApoA1‐mimetic 4F improves M2 polarization and inhibits the function of human macrophages with upregulation of anti‐inflammatory cytokines.^87^ Lappalainen et al.^88^ revealed that the addition of ApoA1 to the macrophage foam cell cultures induces cholesterol efflux and macrophage polarizations. Likewise, HDL prevents TLR4 activation and expression of pro‐inflammatory cytokines with subsequent inhibition of inflammatory signaling pathways, including nuclear factor kappa B (NF‐κB).^89^ TLR4 is mainly associated with cholesterol in lipid rafts and increases macrophages' response to LPS stimulation.^89^ Both native HDL and ApoA1 inhibit the migration of TLR4 to the lipid rafts by depleting cholesterol content.^89^ Similarly, ApoA1 mimetic peptide attenuates the expression of TLR4.^90^ These observations pointed to the protective roles of HDL and ApoA1 against developing inflammatory disorders and abnormal immune responses. However, dysfunctional HDL triggers the development of ED via activation of TLR4/NF‐κB.^91^

Moreover, SR‐B1 inhibits oxidative stress and the release of pro‐inflammatory cytokines from macrophages and contributes to the immunomodulatory effects of HDL.^92^ The macrophages express S1P1 and S1P2 receptors; S1P via S1P1 inhibits the release of pro‐inflammatory cytokines from macrophages and induces macrophage polarization, while S1P2 inhibits macrophage recruitment and migration to the site of inflammation.^93^

Similarly, native HDL and its components, mainly ApoA1, inhibit the maturation and differentiation of dendritic cells DCs) through induction release of IL‐10 and prostaglandin E2, known inhibitors of DCs function and differentiation.^94^ HDL attenuates DCs‐induced T cell activation by inhibiting the release of IL‐12 and TLR4 activation.^94^ It has been shown that the phospholipids fraction of HDL inhibits DCs function and differentiation through modulation of lipid rafts.^94^

In addition, S1P inhibits maturation and differentiation of DCs by increasing IL‐10 with subsequent suppression of Th1 immune response and prompting Th2.^95^ These changes lead to inhibition release of pro‐inflammatory cytokines from DCs with activation release of anti‐inflammatory cytokines. HDL is also regarded as a treatment of chronic inflammatory diseases by suppressing T cells function and proliferation with modulation expression of Th1/Th17. In addition, HDL improves the differentiation of anti‐inflammatory regulatory T cells (Treg) through the polarization of T cells.^66^

These observations suggest the immunological role of HDL and its components, as well as alteration of HDL, may develop during infections and immunological disorders to abnormal HDL with pro‐inflammatory and pro‐atherogenic properties.

**Figure 8:**
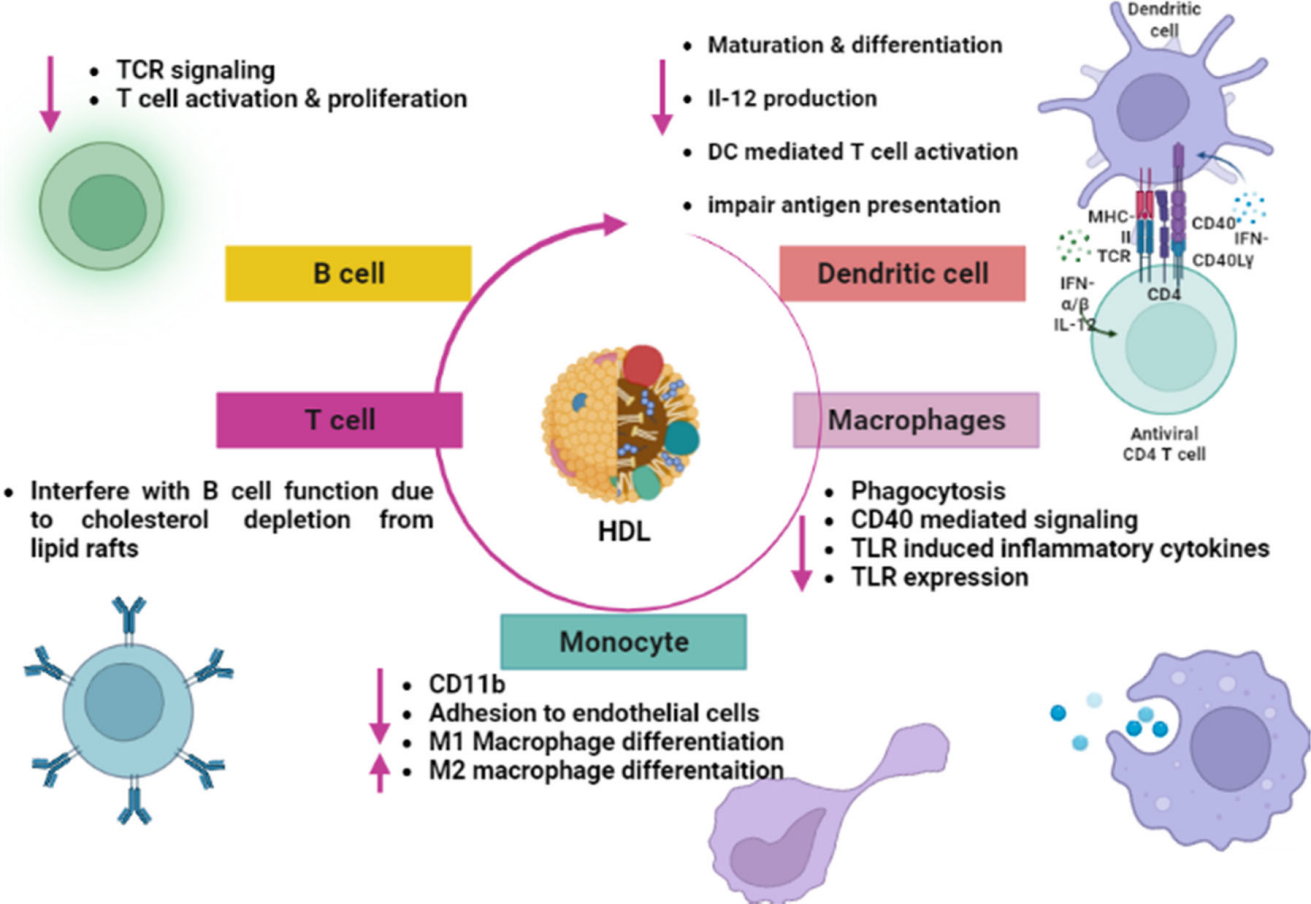
Immunological effects of high‐density lipoprotein.

Therefore, HDL has acute immunological effects on the immune cells, including macrophages, monocytes, DCs, and lymphocytes (Figure 8). Functional HDL inhibits the expression of adhesion molecules, monocyte infiltration, oxidase enzyme and activates eNOS, though dysfunctional HDL acts reversely.

## 3 DYSFUNCTIONAL HDL AND COVID‐19

### 3.1 Native HDL and SARS‐CoV‐2 infection

**Figure 9:**
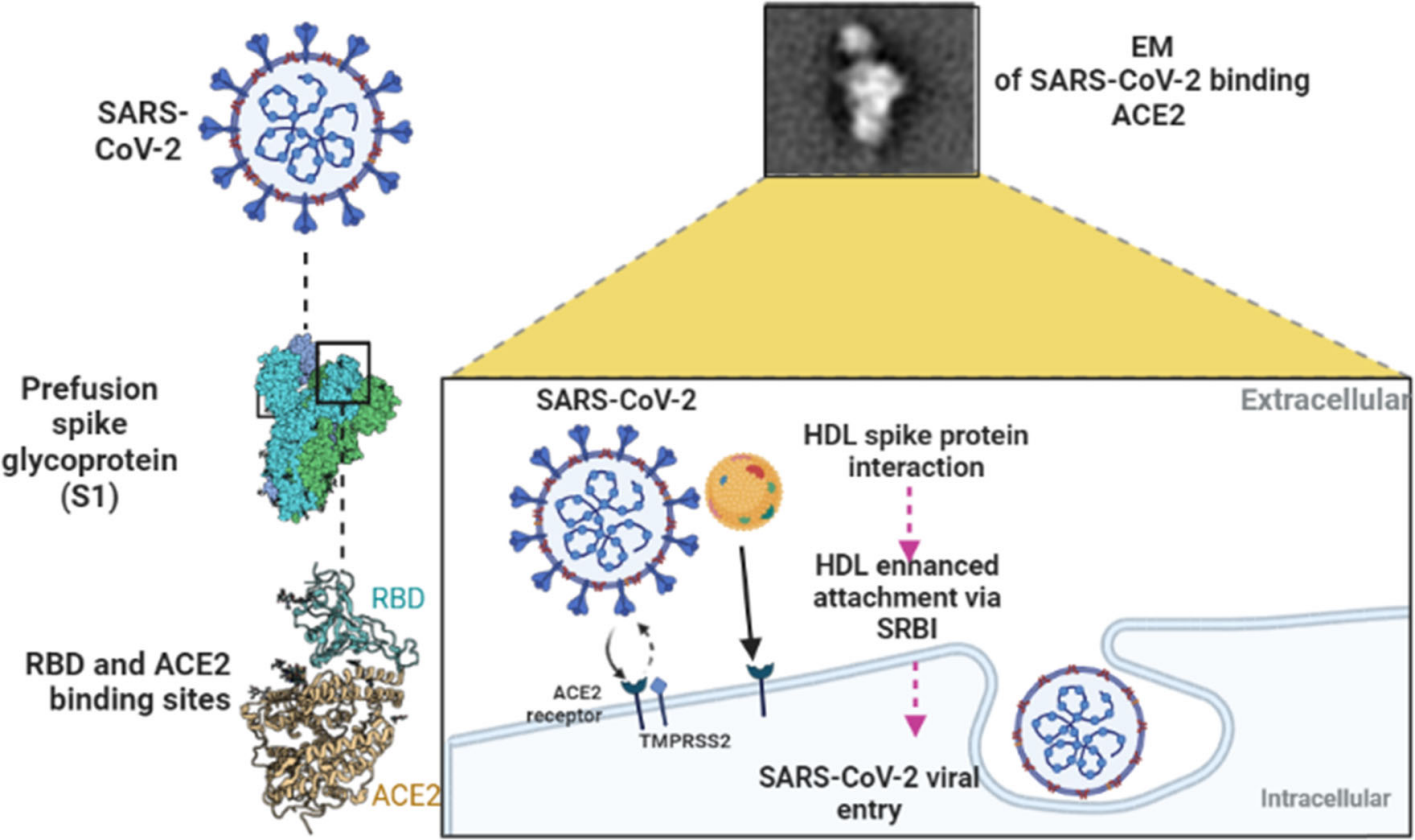
The potential role of high‐density lipoprotein in SARS‐CoV‐2 infection.

Recent findings illustrated that cholesterol plays a critical role in viral replication and internalization of SARS‐CoV‐2 with immune activation in Covid‐19 patients.^96^ In particular, lipids are fundamental components of SARS‐CoV‐2 engaged with a viral membrane fusion with the host cells, endocytosis, viral replication, and exocytosis.^96^ In addition, SARS‐CoV‐2 binds HDL and complex attached co‐localized receptors (ACE2/SR‐B1), which facilitate viral entry (Figure 9). S1 subunit of SARS‐2‐S binds to cholesterol and possibly to HDL components to enhance viral uptake in vitro.^97^ SR‐B1 expression facilitates SARS‐CoV‐2 entry into ACE2‐expressing cells by augmenting virus attachment. Blockade of the cholesterol‐binding site on SARS‐2‐S1 with a monoclonal antibody, or treatment of cultured cells with pharmacological SR‐B1 antagonists, inhibits HDL‐enhanced SARS‐CoV‐2 infection. As well, SR‐B1 is co‐expressed with ACE2 in human pulmonary tissue and in several extra‐pulmonary tissues. Therefore, SR‐B1 acts as a host factor that promotes SARS‐CoV‐2 entry and may help explain viral tropism, identify a possible molecular connection between COVID‐19 and lipoprotein metabolism, and highlight SR‐B1 as a potential therapeutic target to interfere with SARS‐CoV‐2 infection.^97^ In addition, SARS‐CoV‐2 spike protein interferes with the function of lipoproteins, and that this is dependent on cholesterol. In particular, the ability of HDL to exchange lipids from model cellular membranes is altered when co‐incubated with the spike protein.^98^ Also, SARS‐CoV‐2 spike protein removes lipids and cholesterol from model membranes. Thus, spike protein affects HDL function by removing lipids from it and remodeling its composition/structure.^98^ Moreover, HDL is a key component of circulating blood and mainly contains phospholipids, free cholesterol, cholesteryl ester, triglycerides, apolipoproteins, and other proteins. Besides its role in reverse cholesterol transport, HDL displays pleiotropic functions during inflammation and endothelial dysfunction, decreasing inflammatory signaling in immune effector cells and inhibiting endothelial response.^99^ HDL can bind and neutralize viruses and toxic bacterial substances such as lipopolysaccharide (LPS).^100^ Moreover, HDL could block certain viruses to penetrate cells, reducing tissue invasion.^100^ Among HDL mimetic peptides, L‐4F has been more widely employed in several preclinical model of sepsis and has been shown to block production of cytokines, reverse sepsis‐induced hypotension, prevent organ damage, and restore renal, hepatic, and cardiac function, and increase survival rate.^101^ Therefore, HDL seems to plays a double‐sword effect, as native HDL prevents SARS‐CoV‐2 infection and related complications, and in the same time facilitates SARS‐CoV‐2 entry.

Thus, dyslipidemia during SARS‐CoV‐2 infection could be diagnostic and prognostic tools for evaluating response to the clinical therapy. A retrospective study involving 55 hospitalized Covid‐19 patients compared to matched healthy controls showed that HDL and cholesterol levels were reduced in Covid‐19 patients compared to the controls (*p* < .0001).^96^ These findings pointed out that reduction of HDL might due to SARS‐CoV‐2 infection as an epiphenomenon.

In vitro study revealed that SARS‐CoV‐2 via spike protein can bind HDL and facilitate its binding to the ACE2.^102^ Similarly, cell line study by Hernrich et al.^103^ demonstrated that low HDL concentration promotes SARS‐CoV‐2 entry, while high HDL concentration impedes this process. Also, targeting of SR‐B1 and cholesterol suppress entry of SARS‐CoV‐2 in cell culture.^103^ These observations indicated that HDL and its receptors have a potential role in SARS‐CoV‐2 infection.

HDL has potent anti‐inflammatory effects, reducing the release of pro‐inflammatory cytokines and activation of immune cells, including macrophages, DCs, and T cells.^94^ Therefore, HDL may reduce inflammatory and exaggerated immune response as well as progression of cytokine storm in Covid‐19.^104^ In addition, HDL reduces activation of adhesion molecules and neutrophil diapedesis,^105^ which attenuates neutrophil hyper‐activation and development of neutrophil extracellular traps and immunothrombosis in Covid‐19.^105^ Likewise, HDL has anti‐SARS‐CoV‐2 antioxidant effects by its component PON‐1 and SR‐B1,^59^, ^74^ thereby attenuating oxidative stress injury in Covid‐19.^106^

### 3.2 Dysfunctional HDL and SARS‐CoV‐2 infection

In SARS‐CoV‐2 infection, high pro‐inflammatory cytokines and oxidative stress may induce oxidation of HDL, causing elevation of oxidized HDL (oxHDL) serum level, structural and functional instability of HDL with development of dysfunctional HDL.^102^ Furthermore, it has been demonstrated that oxHDL induces cytotoxicity, oxidative stress and inflammation by activating the release of matrix metalloproteinase 9(MMP‐9) and TNF‐α from macrophages through NADPH oxidase‐dependent mechanism.^107^

Begue et al.^108^ revealed that HDL is highly altered in patients with Covid‐19, characterized by reduced ApoA1 and PON‐1 activity. Besides, S1P and ApoM‐HDL are significantly reduced in patients with severe Covid‐19 compared to the controls.^109^ S1P is inversely correlated with Covid‐19 severity, and significantly correlated with CRP, D‐dimer and ferritin, biomarkers of Covid‐19 severity.^109^ The reduction of S1P in Covid‐19 could be due to a reduction in the biosynthesis of ApoM and albumin by the liver that are transporters of S1P or a reduced number of erythrocytes which are the main source of circulating S1P.^109^ It has been reported that 60% of circulating S1P is bound to ApoM of HDL.^110^ The reduction of S1P increases the risk of lymphopenia, a hallmark of Covid‐19 since SIP improves the egress and delivery of lymphocytes from lymphoid organs.^111^ Plasma SIP has anti‐inflammatory and can prevent ED. However, interstitial S1P increases local inflammation and antagonizes the effects of circulating anti‐inflammatory one.^112^ Fingolimod, an antagonist of S1P had been proposed to effective therapy in Covid‐19 by reducing interstitial S1P, but was stopped because of severe lymphopenia.^113^ Besides, glucocorticoids which are widely used in Covid‐19 inhibit the development of interstitial S1P‐induced cytokine storm.^114^ Taken together, reduction of S1P may reduce the functional activity of native HDL with the development of dysfunctional HDL. S1P through S1PR1 induce transmembrane protein serine 2 (TMPRR2), which activates the expression of ACE2. In turn, ACE2 activates the synthesis of S1P by activating sphingosine kinase1/2(Sphk1/2).

Indeed, the concentration of SAA in HDL is increased in patients with severe Covid‐19 that blunt HDL's anti‐inflammatory and antiapoptotic effects.^108^ Therefore, SAA‐bound HDL might be useful as a possible biomarker of Covid‐19 severity.^108^ It has been reported that the composition and function of HDL are highly modified in sepsis and endotoxemia, causing the development of dysfunctional HDL.^57^ Sharma et al.^115^ found that pneumonia is linked with reduction of ApoA1, PON‐1, and HDL with an elevation of acute‐phase proteins including CRP and SAA.

ApoA1 of HDL is declined in Covid‐19 patients due to reduction of hepatic synthesis of ApoA1 through inhibition of ApoA1 gene or replacement of ApoA1 by SAA in HDL.^116^ Synthesis of SAA is augmented during acute inflammatory conditions that replace ApoA1 in HDL.^116^ These changes favor the development of dysfunctional HDL since HDL isolated from Covid‐19 demonstrated a blunted afforded and protective effect against TNF‐α‐induced apoptosis in cell cultures.^108^ A previous study revealed that hepatitis B virus infection (HBV) inhibits the secretion of ApoA1 from the liver causing abnormal HDL.^117^ Recently, Coelho et al.^118^ ApoA1 depletes lipid raft and can neutralize viral nonstructural protein in dengue virus infection. Therefore, ApoA1 mimetic peptide 4F could effectively treat dengue virus infection. Kelesidis et al.'s^119^ in vitro study showed that ApoA1 mimetic peptide 4F could inhibit SARS‐CoV‐2 replication and associated inflammation, apoptosis, and oxidative stress. These observations indicated that dysfunctional or depleted ApoA1 in Covid‐19 may increase SARS‐CoV‐2 replication and high inflammatory complications.

Moreover, LCAT is reduced in Covid‐19 due to SARS‐CoV‐2 infection‐induced hyperinflammation that alters HDL's anti‐inflammatory function.^120^ Ex‐vivo administration of LCAT increases HDL‐bound ApoA1 and reduces HDL‐bound SAA.^120^ These findings proposed that LCAT treatment could benefit Covid‐19 by improving HDL function and preventing the development of dysfunctional HDL induced by SARS‐CoV‐2 and linked associated disorders.

In addition, lipoprotein lipase (LPL) or its regulatory proteins like ApoCII are inhibited during SARS‐CoV‐2 by high pro‐inflammatory cytokines.^121^ Low LPL inhibits triglyceride metabolism, causing high triglyceride and low HDL. Therefore, high triglyceride and low HDL predict the severity of Covid‐19.^121^ Besides, CETP is also attenuated in SARS‐CoV‐2 infection leading to a reduction in biosynthesis and function of HDL.^121^ Inhibition of CETP was observed in patients with hemorrhagic fever and renal syndrome leading to a significant reduction of HDL.^122^

Of note, native HDL has an antiviral effect against SARS‐CoV‐2, while glycated HDL loses its antiviral effects.cLarger HDL with high PON‐1 activity has potent antiviral effects compared to the glycated HDL.^123^ Thus, patients with co‐morbidities like hypertension and diabetes mellitus had low PON‐1 activity increasing their vulnerability for severe Covid‐19.^124^ ApoAII inhibits the interaction between HDL and SR‐B1, displaces ApoA1 and impairs HDL function; it is regarded as an atherogenic factor and promotes pro‐inflammatory cytokines.^62^ Therefore, ApoAII is increased in Covid‐19 patients, causing dysfunctional HDL development with immunoinflammatory complications.^125^

### 3.3 Dysfunctional HDL and risk of thrombosis in Covid‐19

In Covid‐19, PON‐1 is reduced due to oxidative stress and reduction of body antioxidant capacity. As well, high plasmin and elastase from activated neutrophils in Covid‐19 may induce degradation of HDL PON‐1 as well as thrombus formation.^126^ Interestingly, native HDL has antithrombotic action and prevents ED by upregulating endothelial eNOS and prostacyclin by SR‐B1.^127^ In addition, native HDL also reduces platelet aggregations and promotes fibrinolytic pathway.^127^ However, dysfunctional HDL loses these properties and becomes more atherogenic for induction of thrombosis.^128^ Moreover, oxHDL activates SR‐B1 on platelets leading to platelet hyperreactivity and thrombosis.^107^, ^129^ These findings suggest that dysfunctional HDL in Covid‐19 due to pro‐inflammatory cytokines may cause pulmonary thrombosis, a hallmark of Covid‐19 severity.^130^

### 3.4 HDL and inflammatory signaling pathway in Covid‐19

Noteworthy, exaggerated inflammatory signaling pathways in Covid‐19 may interact with HDL bidirectionally. In patients with unstable angina oxHDL and oxLDL trigger the release of NF‐κB with subsequent release of pro‐inflammatory cytokines.^131^ Activated NF‐κB is exaggerated in Covid‐19 and linked with the development of ALI/ARDS.^132^ In turn, NF‐κB‐induced pro‐inflammatory promotes the development of oxHDL. These findings suggest that dysfunctional HDL may trigger an abnormal immune response in Covid‐19 patients.

As well, oxHDL triggers activation of nuclear receptor pyrin 3 (NRP3) inflammasome, which induces the release of IL‐1β, IL‐18, and caspase‐1.^133^ However, native HDL inhibits the expression of NRP3 inflammasome in a dose‐dependent manner.^133^ Besides, NRP3 inflammasome is activated in Covid‐19, causing exaggerated immunoinflammatory response with development of complications.^134^ These findings indicated that dysfunctional HDL could increase Covid‐19 severity by inducing NRP3 inflammasome. As well, exaggerated NRP3 inflammasome induces the development of dysfunctional HDL^135^ in a vicious cycle. Therefore, targeting of NRP3 inflammasome or using antioxidants to prevent the development of oxHDL could effective strategy against Covid‐19.

Of interest, HDL from healthy subjects restores the function of oxLDL by inhibiting mitogen‐activated protein kinase (MAPK).^136^ Though, dysfunctional HDL cannot restore the function of oxLDL but also induce activation of MAPK, causing the release of pro‐inflammatory cytokines.^136^ These observations pointed out that dysfunctional HDL may aggravate Covid‐19 severity by inducing MAPK signaling pathway, which is highly activated during SARS‐CoV‐2 infection.^137^

Amusingly, CD147 is regarded as one of the most important receptors for entry of SARS‐CoV‐2.^138^ Yang et al.^139^ found that oxHDL and oxHDL increase the expression of CD147. These reports and studies proposed that dysfunctional HDL may increase risk of SARS‐CoV‐2 infection and its severity. Moreover, AGE is augmented in SARS‐CoV‐2 infection due to the glycation of lipids and proteins. As well, receptors for AGE (RAGEs) which are highly expressed in pulmonary alveolar cells, are linked with SARS‐CoV‐2‐induced ALI and hyper inflammation.^140^ Thus, AGE/RAGEs are involved in the pathogenesis of Covid‐19. Zhou and colleagues revealed that high AGE/RAGEs are associated with reducing HDL antioxidant potential^141^ and promoting the progression of dysfunctional HDL. These findings highlighted that exaggerated AGE/RAGEs in Covid‐19 could be a possible cause for the development of dysfunctional HDL.

### 3.5 Dysfunction HDL and AngII in Covid‐19

Interestingly, there is close interaction between HDL and AngII as HDL inhibits vascular inflammation by reducing the expression of AT1R,^142^ indicating a protective role of HDL against AngII‐induced inflammation. Wolf and colleagues illustrated that high circulating AngII down‐regulate SR‐B1 in proximal renal tubular cells.^143^ In Covid‐19, SARS‐CoV‐2‐induced dow‐regulation of ACE2 may augment AngII circulation and associated inflammatory disorders.^144^ Therefore, high AngII in Covid‐19 could be a potential reason for the development of dysfunction HDL by inhibiting the protective role of HDL through hnteraction with SR‐B1.

Remarkably, SR‐B1 facilitates entry of SARS‐CoV‐2 into permissive cells, mainly in pulmonary alveolar cells, since SR‐B1is co‐localized with ACE2.^145^ Herein, SARS‐CoV‐2 and high AngII in Covid‐19 may induce dysfunctional HDL by attenuating HDL‐SR‐B1 interaction. As a result, induction expression of SR‐B1 by statins may improve functional HDL^146^ and lead to beneficial effects against Covid‐19 through this pathway.^147^

### 3.6 Dysfunction HDL and vitamin E in Covid‐19

Moreover, HDL is the major source of Vitamin E for pulmonary alveolar type II due to the higher expression of SR‐B1. The deficiency of Vitamin E may deregulate the expression of SR‐B1.^148^ Supplementation of Vitamin E improves HDL's antioxidant and anti‐inflammatory effects.^149^ Of note, Vitamin E is mainly transported by HDL and increases the resistance of HDL against oxidative stress.^150^ Therefore, dysfunctional HDL may reduce Vitamin E's transport and biological function with subsequent development of oxidative stress.^150^ In Covid‐19, serum Vitamin E levels are reduced due to SARS‐CoV‐2‐induced oxidative.^151^ Reduction of Vitamin E in Covid‐19 could be a possible cause for the development of dysfunctional HDL, which in turn aggravates this condition. Therefore, Vitamin E supplementation may improve HDL function by inhibiting oxidative stress.^152^

### 3.7 Dysfunction HDL and hyperferritinemia in Covid‐19

Furthermore, hyperferritinemia, the cardinal biomarker of immune deregulation in Covid‐19, may associate with dysfunctional HDL development.^153^ Hyperferritinemia is adversely affecting HDL function in Covid‐19.^153^ HDL via SR‐B1 increases iron retention in macrophages with subsequent biosynthesis of ferritin.^154^ Besides, high IL‐6 in SARS‐CoV‐2 infection induces hepcidin expression, which also reduces HDL function by reducing ApoA1.^155^ Therefore, the ferritin/hepcidin axis should be concerned with developing dysfunctional HDL.

**Figure 10:**
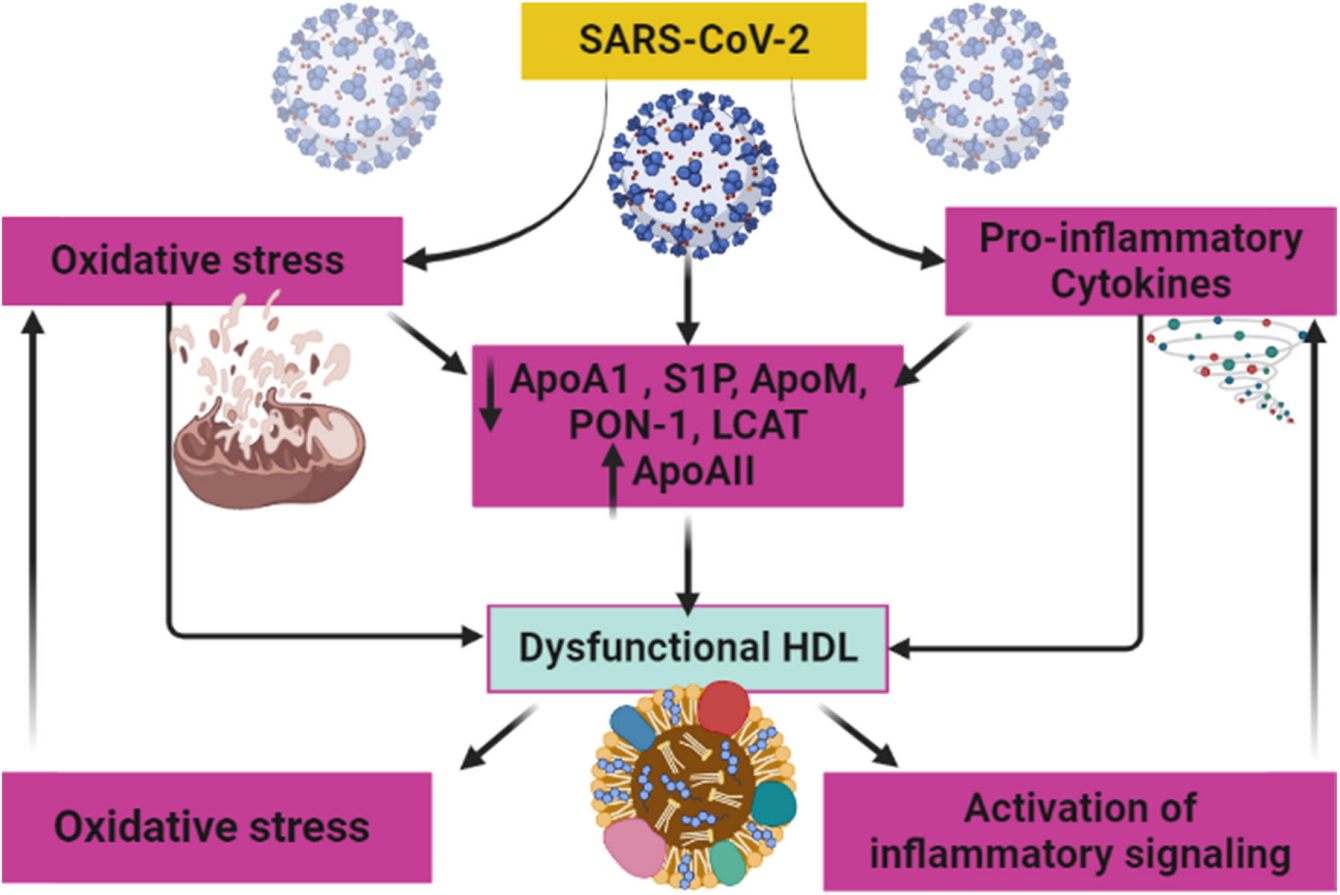
Development of dysfunctional high‐density lipoprotein in SARS‐CoV‐2 infection.

Taken together, it would be important to decide whether dysfunctional HDL seen in Covid‐19 patients may alter SARS‐CoV‐2 infection by promoting viral entry. Further experiments are required to determine the mechanism of SARS‐CoV‐2 infection‐induced dysfunctional HDL, and how dysfunctional HDL affects Covid‐19 severity. Therefore, SARS‐CoV‐2 infection may induce the development of dysfunctional HDL through different mechanisms, including induction of inflammatory and oxidative stress and activation of inflammatory signaling pathways. In turn, dysfunctional HDL through induction expression of inflammatory signaling pathways and oxidative stress may increase Covid‐19 severity (Figure 10).

## 4 CONCLUSIONS

Native HDL has anti‐inflammatory and antioxidant effects. In SARS‐CoV‐2 infection, high oxidative stress and pro‐inflammatory cytokines may induce development of dysfunctional HDL, which in turn triggers oxidative stress and more pro‐inflammatory cytokines. Therefore, dysfunctional HDL is implicated in the pathogenesis of SARS‐CoV‐2 infection and may increase Covid‐19 severity. Thus, inhibiting inflammatory and oxidative stress disorders in Covid‐19 may attenuate the development of harmful dysfunctional HDL. Experimental, preclinical, and clinical studies are recommended to elucidate the potential role of HDL in SARS‐CoV‐2 infection, and how dysfunctional HDL is developed.

## AUTHOR CONTRIBUTIONS

**Hayder M. Al‐kuraishy**: Conceptualization (equal); writing—original draft (equal); formal analysis (equal). **Nawar R. Hussien**: Writing—original draft (equal); formal analysis (equal). **Marwa S. Al‐Niemi**: Writing—original draft (equal); formal analysis (equal). **Esraa H. Fahad**: Methodology (equal); writing—review and editing (equal). **Ali K. Al‐Buhadil**y: Writing—original draft (equal); formal analysis (equal). **Ali I. Al‐Gareeb**: Writing—original draft (equal); formal analysis (equal). **Sadiq M. Al‐Hamash**: Methodology (equal); writing—review and editing (equal). **Christos Tsagkaris**: Methodology (equal); writing—review and editing (equal). **Marios Papadakis**: Methodology (equal); writing—review and editing (equal). **Athanasios Alexiou**: Methodology (equal); writing—review and editing (equal). **Gaber El‐Saber Batiha**: Conceptualization (equal); methodology (equal); writing—review and editing (equal). All authors read and approved the final manuscript.

## CONFLICT OF INTEREST STATEMENT

The authors declare no conflict of interest.

## Data Availability

The data that support the findings of this study are available from the corresponding author upon reasonable request.
